# Examination of differential ratings of perceived exertion (dRPE) during bio-banded small-sided games

**DOI:** 10.1371/journal.pone.0270259

**Published:** 2022-07-29

**Authors:** Steve Barrett, Kieran Short, Alex Lowthorpe, Paul Swinton, Patrick Maughan, Ally Hamilton, Frances Hunter, Chris Towlson

**Affiliations:** 1 Playermaker, London, United Kingdom; 2 Department of Sport, Health and Exercise Science, University of Hull, Hull, United Kingdom; 3 School of Health Sciences, Robert Gordon University, Aberdeen, United Kingdom; 4 School of Life Sciences, University of Glasgow, Glasgow, United Kingdom; 5 Hull City AFC, Hull, United Kingdom; 6 Middlesbrough Football Club, Middlesbrough, United Kingdom; Universidade Federal de Mato Grosso do Sul, BRAZIL

## Abstract

The aims of the current study were to investigate the use of dRPE with academy soccer players to: 1) examine the effect of bio-banded and non-bio-banded maturity groups within SSG on players dRPE; 2) describe the multivariate relationships between dRPE measures investigating the sources of intra and inter-individual variation, and the effects of maturation and bio-banding. Using 32 highly trained under (U) 12 to U14 soccer players (mean (SD) age 12.9 (0.9) years, body mass 46.4 (8.5) kg and stature 158.2 (14.9) cm) academy soccer players from two English professional male soccer academies. Players were categorised according to somatic maturity status using estimated percentage of adult stature attainment, with players randomly assigned into teams to play 4v4 SSG. The study used a repeated measures design, whereby the selected players participated within 6 bio-banded (maturity matched [pre-PHV Vs pre-PHV and post-PHV vs post PHV] and miss-matched [pre-PHV vs post-PHV] and 6 mixed maturity SSG at their respective clubs. Using mixed and fixed effect regression models, it was established hat pre-PHV players exhibited higher dRPE compared with their post-PHV counterparts. Mixed bio-banded games reported higher dRPE outputs overall. Variation in dRPE measures across a series of bio-banded games are caused by both between and within sources of variation in relatively equal amounts. Across a series of bio-banded games, the four dRPE measures do not provide unique information, and between variation is best expressed by one or two highly correlated components, with within variation best explained by a single equally loaded component. Using a bio-banding SSG design study, we have shown that pre-PHV players report higher subjective measures of exertion than post-PHV players during. Additionally, when evenly mixing players based on measures of maturation, higher measures of perceived exertion were generally reported.

## Introduction

The highly individualized, non-linear relationship between age and the development of maturity-related anthropometric characteristics (e.g., stature and body-mass) [1, 2] often leads to the over-selection of early maturing soccer players for academy development programs in comparison to their later maturing counterparts [3–5]. This selection bias is attributed to early maturing players who are often characterized as being temporarily taller and heavier, but not necessarily technically better, in comparison to their later maturing team-mates [3]. Therefore, soccer match and training formats which can control for the confounding effect of biological maturation [4–6] are of importance and relevance to player development practitioners responsible for prescribing suitable training loads and playing environments.

The grouping of athletes according to their maturation status, rather than chronological age is commonly referred to as bio-banding [4, 5, 7]. The exclusive [8] objective of bio-banding is to reduce between player maturity-related differences in anthropometric [9], and physical fitness characteristics, and create a more equitable playing and training environment [10–17]. Bio-banding has been shown to be an effective strategy to reduce the large within group variations associated with the early development of maturity-related anthropometric characteristics, such as stature and body-mass [1, 2] which can be directly associated to the maturation process [9]. Typically, bio-banding methods use one of two popular methods for estimating maturity status [18], including maturity offset methods [19–21] (i.e. estimating the number of years from/past the onset of peak height velocity [PHV]), or estimating the players percentage of final adult stature [22]. Bio-banding has become increasingly popular within academy soccer practices to enhance practitioner understanding of the influence player maturity status has on player characteristics including psycho-social [10, 12], technical-tactical [10, 11, 14], and perceived effort [10, 14]. Which are all considered important by talent practitioners [23, 24] during soccer match-play. Not only is the application of bio-banding important to key stakeholders, such as parents or guardians, and coaches responsible for supporting, and the (de)selection of players for development programs [25], but the consideration of player maturation status is of relevance to practitioners who are responsible for the prescription of training loads and volumes [26]. This concern is evidenced by academy soccer players who have been categorized as being pre-PHV accumulating greater session ratings of perceived exertion training load (sRPE-TL) (being RPE*activity duration in minutes) when competing in small-sided games (SSG) against their more mature (i.e. post-PHV) counterparts, despite internal measures of training load suggesting no meaningful difference between the groups of players [10].

Training loads within soccer are commonly assessed with different metrics that tend to be grouped by internal (e.g. heart rate, blood lactate) or external training loads (e.g. total distance covered, high speed running distances etc.) [27, 28]. Within youth soccer, measurements of external training loads have focused on time-motion analysis data [29, 30], typically reported from micromechanical electrical systems (MEMS) devices or more commonly referred to as global positioning systems (GPS) devices. In contrast, measurement of internal training loads have focused on heart rate and subjective load data [28]. Rather than providing distinct information, it is likely that measures of internal and external training load will demonstrate various associations and relate to other constructs such as intensity and volume of training [31, 32]. In research conducted with academy soccer players, Maughan et al. (2021) used principal component analysis (PCA) to investigate the underlying structure of the relationships between external measures of training load and sRPE-TL. Their results identified that most of the variation within the data could be explained by two components reflecting the training volume (to which sRPE-T) and training intensity [33]. The authors concluded that multivariate techniques should be employed to better understand the complex nature of training loads in youth soccer. Which is of relevance to academy practitioners, given that the highly individualized effect of biological maturation on players anthropometric (i.e. stature and body-mass) development [1, 2] has been associated with subsequent temporary alterations in soccer players functional movement capacity [34]. Such alterations may lead to imbalances between strength and flexibility, and temporary reductions in movement mechanics typically referred to as “adolescent awkwardness” [35]. Ultimately, leading to increased risk of sustaining a non-contact injury [36]. An extension of this conclusion holds that multivariate techniques may enable more effective monitoring and subsequent management of training loads in academy soccer players who are undergoing PHV.

In addition to delineating between training volume and intensity, it has been suggested that perceptual measures to quantify the contributions of specific cardiovascular and neuromuscular/musculoskeletal systems can provide relevant information to monitor and prescribe training in team sports [37, 38]. Differential ratings of perceived exertion (dRPE) that separate scores for breathlessness (RPE-B), leg muscle exertion (RPE-L), and technical/cognitive exertion (RPE-T) [38] may provide a viable alternative to quantify internal loads [39, 40]. To date, there has been no research investigating the full range of dRPE measures within an academy soccer context. However, studies conducted using Australian Football League have shown there to be likely small differences between RPE-L and RPE-T (5.5%), a likely small difference between RPE-L and RPE-B (3.5%) and a possibly small difference between RPE-B and RPE-T (1.9%) [38]. With combined dRPE training loads explaining 66–91% of the variance in sRPE training loads within professional rugby union players, and the strongest associations being exhibited with sRPE-TL being sRPE-L for high-intensity intervals (*r* = 0.67), sRPE-B for repeated high-intensity efforts (*r* = 0.89) and sRPE-T for Speed (*r* = 0.63) and Skills (*r* = 0.51) [37]. Additionally, most research investigating dRPE has used only correlation analyses that are limited in their ability to explore underlying relationship structures, with data comprising more than two variables. Whilst PCA provides a more effective method to explore structures [31], the method has limitations when applied to repeated measures data, where many series of measurements are made across the same variables for a relatively small number of individuals. In contrast, more modern multivariate techniques, including MultiLevel Simultaneous Component Analysis (MLSCA) can better investigate both the sources of inter- and intra-individual variability in multivariate data and identify individuals or groups of individuals that differ with regards to measures of location, variance and covariance [41]. In the context of team sports, MLSCA can be used to identify whether the variability in multivariate measurements are primarily caused by differences across individuals, or differences within individuals due to variation in relevant interventions or independent variables. Additionally, MLSCA can be used to identify individuals who tend to score highest/lowest or are the most/least variable on multivariate components described by combinations of the outcome variables [42]. Given the popularity of bio-banding methods and the increased focus on multivariate statistical techniques, the aims of the current study were to investigate the use of dRPE with academy soccer players to: 1) examine the effect of bio-banded and non-bio-banded maturity groups within SSG on players dRPE; 2) describe the multivariate relationships between dRPE measures investigating the sources of intra and inter-individual variation, and the effects of maturation and bio-banding.

## Methods

Having institutional ethics committee approval (FHS189) and parental/guardian consent, the current study used previous methods [16] which have investigated the perceived exertion of academy soccer players during SSG`s, when teams were selected based upon their chronological age or their bio-banding grouping. Additionally, this study investigated if dRPE measures provide distinct information, and to establish the associated sources of variation including between and within players.

### Participants

Thirty-two highly trained under (U) 12 to U14 male soccer players (mean (SD) age 12.9 (0.9) years, body mass 46.4 (8.5) kg and stature 158.2 (14.9) cm) from two, English professional soccer academies were invited to participate in the study. Players were categorised according to somatic maturity status using estimated percentage of adult stature attainment EASA [22], with players randomly assigned into teams to play 4v4, 5 minute SSGs`. The study used a repeated measures design, whereby the selected players participated within 6 bio-banded (maturity matched [pre-PHV Vs pre-PHV and post-PHV vs post PHV] and miss-matched [pre-PHV vs post-PHV]), and 6 mixed maturity SSGs at their respective clubs.

#### Anthropometric and maturity measurements

Using previously used methods [1, 3, 6, 43], each player’s anthropometric (stature and body-mass) characteristics were recorded. These measures were used in conjunction with adjusted [44], self-reported mid-parental stature of both biological parents of the player to provide an estimated percentage of adult stature (%EASA). This method was selected due to its enhanced ability to correctly identify the onset of PHV in academy soccer players in comparison to maturity offset-based measures [45]. In accordance with our previous work [10], we defined our bio-bandings groupings as ‘*post*-PHV’ (≥90% EASA) (n = 16) and ‘*pre*-PHV’ (<90% EASA) (n = 16). Whilst we acknowledge that PHV has been shown to occur at approximately 86% of estimated adult stature [12], bandings were defined as ‘post-PHV’ (≥90% EASA) and ‘pre-PHV’ (<90%) to allow even distribution of players per category. Given that previous research has shown bio-banding to have little effect on players within the circa-PHV category [10, 15, 16], only players at either extreme of the maturation continuum were selected to participate in the study. Players anthropometric data was collated within the month prior to the testing period, extenuating the influence of biological growth on subsequent accurate maturity bandings. Using the aforementioned Khamis and Roche [23] method, players were assigned into one of four ‘bio-banded’ teams, two teams of four post-PHV (n = 8) maturing players and two teams of four pre-PHV (n = 8) maturing players and contested both maturity-matched and miss-matched SSGs using a ‘round-robin format’. On completion of these the bio-banded SSGs, players received 20 min passive recovery before being randomly and independently (i.e. no prior knowledge regarding each player’s somatic characteristics) allocated to 4 mixed-maturity (comprised of 2 ‘pre’ and 2 ‘post-PHV’ players) teams to act a surrogate control measure [10] and repeat the same ‘round-robin’ format of SSGs. To permit statistical comparison, mixed-maturity teams were aggregated in to two ‘mixed’ maturity bandings (e.g. team 1A and 1B were aggregated to form group A) to permit pairwise comparisons.

#### Small-sided games

In both conditions, players performed a standardised, 15-minute club led warm-up and contested 6 bio-banded and mixed maturity SSGs (total: n = 6) on an outdoor 4G surface, The SSGs were contested by teams of four players. This method has been previously used for the intention of talent identification within UK soccer academy practice [46] and previous bio-banding SSG research [10, 15, 16]. In addition, given the prevalence of the maturation selection bias with UK academy soccer programmes [3, 43] the sample of players per academy deemed to be pre-PHV was finite. Therefore, to permit a maturity matched (e.g., Pre-PHV vs pre-PHV; post-PHV vs post-PHV) SSG format, it was considered intuitive to implement teams comprised of four players. Matches were contested on a 24 m x 24 m (576 m^2^; 72 m^2^per player), with the relative pitch size being consistent throughout the duration of the specific testing condition. This pitch size was selected as previous research, using a pitch size of 18.3m x 23.0m (52m^2^ per player), highlighted the smaller pitch size as a limiting factor when assessing maturity-related outputs during bio-banded SSG [10]. Given the paucity of bio-banding research that has used SSG formats [10, 15, 16], and that the aim of the study was to examine the effect of bio-banding and not the influence of relative pitch size per *se* on players dRPE, a pitch which was considered representative of ‘normal’ playing conditions and familiar to the players was implemented. Each SSG was interspersed with a three-minute interlude of passive recovery [46]. As per previously used methods [10, 12, 46, 47], two goals (2 m x 1 m) with no identified goalkeepers were applied, goals were only permitted to be scored from a position within the attacking half of the pitch, whilst using a multi-ball system to encourage continuous and flowing match-play. Club staff were reframed from providing any verbal feedback or encouragement to players throughout the duration of the session. Each team would receive a minimum of five and a maximum of fifteen minutes of low intensity, active recovery between SSG’s, in which players performed club specific, standardised technical drills to maintain match readiness and reduce tedium.

### Differential Ratings of Perceived Exertion (dRPE)

After each bio-banded and mixed SSG, players recorded a gestalt RPE score which was multiplied by session duration (sRPE-TL), alongside scores for breathlessness (sRPE-B), leg muscle exertion (sRPE-L), and technical demand (sRPE-T). Scores were recorded individually using a numerically blinded CR100^®^ scale [47] via a custom-built mobile application running on a 7” Android tablet (Iconia One 7 BI-750, Taipei Twaiwan: Acer Inc.) [37]. The CR100^®^ scale was chosen over the more commonly used CR10^®^ RPE as the scales finer grading has potential to provide a more sensitive appraisal of exertion in soccer players [48]. Each player was familiar with the scale and the recommended researcher instructions for scale administration were used [49]. Specifically, the players were prompted with the following screen text for each dRPE measurement: *“Using the verbal expressions on the scale below*, *please rate your (individual dRPE measure) perception of exertion for the match”*. Each dRPE measurement was individually shown on the screen, with a sliding scale to mark the appropriate word/ line to describe their exertion. Players were separated to ensure anonymous scores were provided without the influence of other players by having two tablets (one per team), with each team forming a line away from the player completing the form. Players were only included in the analysis of dRPE if they had completed dRPE post all their SSG.

### Statistical analysis

Potential effects of bio-banding and maturation on dRPE values were first analysed by conducting mixed effect regression models, with random effects included for individual players to account for the repeated measures nature of the data, and fixed effects included for bio-banding groupings (pre-PHV vs post-PHV), game type (bio-banded vs mixed) and the interaction between the two variables. The extent to which main effects differed from the null value were indexed by *p* values calculated using t-tests on regression coefficients and Satterthwaite’s approximation for degrees of freedom with the lmerTest library in R [50]. Structure of the relationships between dRPE variables was analysed through MLSCA which models the data matrix ***X*** comprising *i* = 1,…,*I* columnwise concatenated data blocks, each consisting of *K_i_* observations of *j* = 1,…,4 dRPE variables. The data matrix is split into three parts including an offset term, a between-part that is used to describe inter-individual variation and a within-part used to describe intra-individual variation in the multivariate data with.

$$x_{ijk_{i}}=x_{ijk_{i}}^{offset}+x_{ijk_{i}}^{between}+x_{ijk_{i}}^{within}=x_{.j.}+(x_{ij.}-x_{.j.})+(x_{ijk_{i}}-x_{ij.})$$

where *x_.j._* is the mean score on variable *j* computed across all data blocks, and *x_ij._* is the mean score on variable *j* computed within data block *i*.

The source of inter-individual variation was computed by performing PCA on the between-part of the data matrix with subsequent varimax rotation to facilitate interpretation. Selection of the number of between components was made using the CHull test [51]. Magnitudes of the between-loadings for each of the components selected were used to interpret the nature of the components and between-scores obtained for each individual after counter rotation. As the between-scores identify systematic differences between individuals according to the multivariate components, the effects of maturation status on these variables were investigated with independent t-tests and calculation of Cohen’s d.

The most general variant to analyse the source of intra-individual variation would be to perform a separate PCA on the within-part of the data matrix for each individual. However, this is likely to lead to problems with interpretation and it should often be expected that within component loadings are similar across individuals [42]. Therefore, the least restrictive variant of MLSCA (MLSCA-P) was employed, creating a single set of within component loadings but enabling variances and correlations of the component scores to vary across individuals [52]. Because $X_{i}^{between}$ and $X_{i}^{within}$ matrices are mutually orthogonal, the parameters of between and within models can be estimated separately, with parameters of the within model obtained via singular value decomposition [52]. As with the between model, magnitudes of the loadings for each of the components selected using the CHull test were used to interpret the nature of the components and the effects of maturation status on individual variance of components was investigated with independent t-tests and calculation of Cohen’s d.

## Results

Descriptive statistics of dRPE values for pre-PHV and post-PHV players are presented in Table 1. Results from mixed effects models (Table 2) identified that in general, pre-PHV players reported higher values for perceived exertion, with point estimates indicating relative increases ranging from ~6 to 16 units (*p* <0.001 to *p =* 0.121). Main effects were also identified for game type, with players reporting higher values (~8 to 9 units) across all RPE variables (*p*<0.001). However, interaction effects between maturity and game type across all RPE variables (*p≤*0.012) identified that increases in RPE values for mixed games were highest for post-PHV players (Table 2).

**Table 1:** Means and standard deviations of dRPE values across groups.

Group	sRPE (±sd)	sRPE-B (±sd)	sRPE-L (±sd)	sRPE-T (±sd)
Pre-PHV	51.2 (15.1)	45.7 (15.3)	44.6 (15.9)	51.7 (14.5)
Post-PHV	42.5 (17.0)	44.2 (14.7)	39.5 (15.6)	41.4 (16.1)

**Table 2:** Results of mixed effects regression models of maturation and game type effects on dRPE values.

	sRPE	sRPE-B	sRPE-L	sRPE-T
Variable	Regression	Regression	Regression	Regression
	Coefficient	Coefficient	Coefficient	Coefficient
Intercept	35.9 (p<0.001)	38.3 (p<0.001)	33.8 (p<0.001)	35.1 (p<0.001)
Pre-PHV	14.4 (p<0.001)	6.2 (p = 0.121)	9.8 (p = 0.019)	15.6 (p<0.001)
Mixed games	8.1 (p<0.001)	7.5 (p<0.001)	8.7 (p<0.001)	9.1 (p<0.001)
Interaction (mixed games & pre-PHV)	-6.4 (p<0.001)	-3.8 (p = 0.012)	-4.3 (p = 0.004)	-6.8 (p<0.001)

Intercept reflects the mean value for early maturation players in bio-banded games. Pre-PHV reflects the change in mean from the intercept for pre-PHV players. Mixed games reflect the change in mean from the intercept for mixed games. Interaction represents the additional change in mean only for both pre-PHV players in mixed games.

The MLSCA analysis identified that variation in dRPE measures were explained relatively equally from between (59.8%) and within (40.2%) sources. CHull tests identified that two components should be selected for the between model, and a single component for the within model (Table 3). For the between model, the first component loaded sRPE-B and sRPE-L, with the second component loading sRPE and sRPE-T (correlation between components equalled 0.82). For the within model, the single component represented equal weighting across all four RPE measures. Adding another component to the within model revealed the same structure that was obtained for the between model, with loadings of sRPE-B and sRPE-L, and loadings of sRPE and sRPE-T (correlation between components equalled 0.87). Use of the MLSCA loadings to generate multivariate perceived exertion values aligned with the univariate regression models, with the between model identifying greater values from post-PHV players (component 1: d = 0.51, *p =* 0.094; component 2: d = 0.95; p<0.0015). In contrast, analysis of the within component variation identified higher mean values for post-PHV players with a medium effect size (d = 0.53; *p* = 0.085).

**Table 3:** MLSCA between- and within-model component loadings and percentage of explained variance.

	Between-model 1 component	Between-model 2 component			Within-model 1 component	Within-model 2 component	
	Component 1	Component 1	Component 2		Component 1	Component 1	Component 2
% of overall variance explained	50.1%	54.9%		% of overall variance explained	27.4%	32.2%	
% of between variance explained	83.6%	91.7%		% of within variance explained	68.0%	80.1%	
sRPE	0.927	0.207	0.765	sRPE	0.804	0.061	0.816
sRPE-B	0.908	1.018	-0.070	sRPE-B	0.834	0.974	-0.077
sRPE-L	0.910	0.932	0.018	sRPE-L	0.844	0.813	0.097
sRPE-T	0.913	-0.094	1.053	sRPE-T	0.816	-0.057	0.949

## Discussion

The current study examined the effect of bio-banding in academy soccer players within 4v4 SSG’s and the influence on players dRPE. The main findings of the study were; 1) In general, pre-PHV players exhibited higher dRPE compared with their post-PHV counterparts; 2) Mixed maturity games reported higher dRPE outputs overall; 3) Variation in dRPE measures across a series of bio-banded games are caused by both between and within sources of variation in relatively equal amounts; 4) Across a series of bio-banded games, the four dRPE measures do not provide unique information, and between variation is best expressed by one or two highly correlated components, with within variation best explained by a single equally loaded component.

Bio-banding in soccer is a method for grouping adolescent players to reduce the within group variations of maturity related anthropometric characteristics [9], which is purported to support the talent identification and player development pathway [10]. Within the current study, those players defined as pre-PHV reported 20–50% higher dRPE values across all constructs for both bio-banded and mixed maturity SSG’s. Despite evidence to suggest maturity-related differences in perceptual effort during SSG match-play exist [9], to date, studies exploring all constructs of dRPE within academy soccer SSG games based upon maturity status are absent. The findings observed when playing against post-PHV are like that observed within senior soccer players, when playing against opponents of a higher rank, with sRPE-T showing large differences [53]. Towlson and colleagues (2021) suggested that players bio-banded as ‘pre-PHV’ had increased challenges to overcome playing against post-PHV players, which would suggest an increase in perceived load consistent with the findings of the current study. Interestingly, during mixed maturity SSG`s, both pre-PHV and post-PHV developers dRPE scores increased. This may be because of accumulated player fatigue, with the players having competed in 60 minutes of SSG match-play. However, given that the players received at least 5 minutes of passive recovery between games, accompanied by an additional 20 minute of passive recovery between the bio-banded and mixed maturity categorisation formats, it is unclear if this elevation in RPE was a direct of result of accumulated fatigue or change in game format. During analysis of passing networks during SSG’s, there is an increased reliance on the post-PHV players during mixed games in comparison to bio-banded games [16]. This subjective reliance was characterised by post-PHV players becoming more integral to passing networks and team dynamics. This was assessed via betweenness centrality, a measure of how often a player lies on the shortest path in the passing network from one player to another, and page rank, a measure of player importance within team dynamics [16]. These findings, alongside those in the current study provide some context and consideration for practitioners considering the practical benefits of using bio-banding during talent identification and development processes. Whilst post-PHV players have been shown to report lower perceived exertion within SSG`s, their involvement appears to be higher than pre-PHV players. Pre—PHV players report higher values of perceived exertion, however, are possibly more reliant on post-PHV players [16] during mixed maturity SSG`s. This could further contribute to the over-selection of early maturing players, who often possess advanced maturity-related anthropometric (primarily enhanced stature) and physical fitness characteristics for key defensive roles (e.g., central defence and goalkeeper) [43, 54]. Subsequently homogenising the type of player within the academy system which a senior squad can select from.

Ratings of perceived exertion have been used to assist academy practitioners to assess the internal load ensued by academy players during chronologically [10] and bio-banded [14] match-play. However, it has been suggested that the one-dimensional approach of overall session ratings of perceived exertion (sRPE) oversimplifies the self-perception of effort [37], suggesting the perception of effort should be differentiated in to specific categories [37]. However, previous research has shown that different dRPE measures may provide similar information when analysed using multivariate methods [33]. When using PCA to investigate relationships between objective and subjective load measures, Maughan et al. [33] found that measures of sRPE, sRPE-L and sRPE-B were heavily loaded within the first principal component that they suggested represented a measure of total training load. Further multivariate analysis through exploratory factor analysis, identified four latent factors, one of which represented subjective load [33]. The authors suggested that univariate correlations, alongside the relationships found in the multivariate assessments suggested that differentiating subjective measures of load had limited benefits for the population analysed. Similar findings were obtained in the present study when analysing the dRPE measures with MLSCA. The analyses identified that both between and within sources were relatively equal (59.8 vs. 40.2%, respectively) in explaining variation in dRPE values. That is, dRPE values were systematically different across players (e.g. due to factors such as maturation), but also varied within players across games (e.g. due to factors such as game type). Statistical tests suggested that a single component model was best to explain with variation, and a two-component model was best to explain between variation. However, the components for the two-component were highly correlated (r = 0.82). When utilising a two-component model, both the between and within-models produced a split between sRPE-B and sRPE-L which loaded heavily within the first component and sRPE and sRPE-T which loaded heavily in the second component. These findings suggest that when taking subjective measures during SSG in academy soccer players, the individuals gestalt sRPE provided similar information to a separate subjective measure assessing technical demands (sRPE-T). Equally these measures may be somewhat distinct to measures of physical exertion regarding breathlessness (sRPE-B) and leg muscle exertion (sRPE-L). The findings that the four dRPE measures did not measure distinct constructs and that they were more likely to represent a single weighted component, or two closely correlated components may be influenced by the data collection environment and its relative homogeneity (e.g., collection of data across very similar SSG’s). The potential for dRPE values and relationships to be influenced by the type of training has previously been argued (26). Given the data collected here was from SSG, placing demands on the technical abilities of players, it is reasonable to expect there to be some relationship between sRPE and sRPE-T. Previous research in rugby union players found relationships between sRPE and sRPE-T for training categorised as ‘skills’, however weaker relationships for ‘skill-based conditioning’, the category which most resembled SSG [37]. Collectively, these findings suggest there is a modality effect on the relationship between dRPE measures, and this is likely to be affected by the participants and the training modality investigated.

Despite this study showing early evidence to suggest that maturity related differences in perceived exertion of players during SSG`s and that bio-banding may be a suitable method to control for this, there are limitations to this study which should be considered. Firstly, data were collected from two English professional male soccer academies and, as per previous research [14], the findings may not be generalisable to other clubs, levels, or sports. Given that the adolescent growth spurt in male academy soccer players typically manifests between 9.7–10.7 years to 13.8–15.2 years [1, 2], methods used for assessing subjective perceptions of exertions were considered appropriate. Previous research using the Borg CR10 scale highlighted a lack of sensitivity which may influence relationships between variables. The CR100 scale, which was used in the present study, has been suggested due to its increased sensitivity in comparison to the CR10 scale [37]. This increase in sensitivity was not evidenced within this study and may be due to factors such as the cognitive maturity of the players which was not measured within the present study. Previous research in youth soccer players has endorsed the use of scales which contain both pictorial and verbal anchors, such as the OMNI scale, to aid players in differentiating between RPE categories [55]. Further research investigating the use of different scales, alongside multivariate methods of analysis, may further the understanding of practitioners with regards to relationships between dRPE measures.

## Conclusion

Using a bio-banding SSG design study, we have shown that pre-PHV players report higher subjective measures of exertion than post-PHV players during SSG`s. Additionally, when evenly mixing players based on measures of maturation, higher measures of perceived exertion were generally reported. Collectively these findings support previous findings by Towlson and colleagues (2021) and further suggest that the use of maturity-matched bio-banded formats create a more equitable (physical) playing environment to supplement academy soccer player development and talent identification pathways. Whilst maturity-matched bio-banded formats may control the exertion experienced by young players, practitioners should also consider that exposure to adversity that comes from being exposed to bigger, stronger and faster players may limit players opportunity demonstrate key psychological behaviours [12] which talent practitioners perceive as most important during the talent identification process [25]. Mixed formats may also promote an over-reliance on early-developed players, leading to higher perceived exertion generally across both post-PHV-, and pre-PHV players [16]. Finally, measures of dRPE were shown to have unique relationships, with gestalt sRPE providing similar information to sRPE-T and measures of sRPE and SRPE-L also showing distinct relationships. Further research may wish to consider whether measures of dRPE within similar populations are training mode dependent, providing further information to support practitioners aims of enhancing talent ID pathways. Therefore, the practical applications of this study are twofold: 1) practitioners should be aware that during SSG match-play pre-PHV players perceive higher levels of exertion than post-PHV maturation players, additionally mixed bio-banded games result in higher reported dRPE values overall, therefore player maturation status must be considered by practitioners during SSG team selection and be influenced by the desired outcome of the intervention. These considerations should create more optimum training environments, thus supporting player development. 2) Whilst collection of dRPE measures may provide useful information with regards to the subjective experience of players, practitioners should be aware that during SSG gestalt sRPE provides similar information to sRPE-T, and similarly sRPE-B and sRPE-L provide similar information. Depending on the aims of the training intervention, practitioners may wish to only consider one, or at most two, measures of subjective load.
